# Meta-Analysis and Systematic Review of Micro- and Macro-Nutrient Intakes and Trajectories of Macro-Nutrient Supply in the Eastern Mediterranean Region

**DOI:** 10.3390/nu13051515

**Published:** 2021-04-30

**Authors:** Radhouene Doggui, Hanin Al-Jawaldeh, Jalila El Ati, Rawhieh Barham, Lara Nasreddine, Nawal Alqaoud, Hassan Aguenaou, Laila El Ammari, Jana Jabbour, Ayoub Al-Jawaldeh

**Affiliations:** 1 Department of Family Medicine, Université de Sherbrooke, Sherbrooke, QC J1K 2R1, Canada; 2 Centre de Formation Médicale du Nouveau—Brunswick, Moncton, NB E1A 3E9, Canada; 3 Health Science Department, The American University of Madaba, Madaba 11821, Jordan; haljawaldeh@yahoo.ca; 4 SURVEN (Nutrition Surveillance and Epidemiology in Tunisia) Research Laboratory, INNTA (National Institute of Nutrition and Food Technology), Tunis 1007, Tunisia; jalila.elati@yahoo.fr; 5 Nutrition Division, Department of Non-Communicable Disease, Ministry of Health, Amman 11118, Jordan; majeda_barham@hotmail.com; 6 Department of Nutrition and Food Science, American University of Beirut, Beirut 11-0236, Lebanon; ln10@aub.edu.lb; 7 Food and Nutrition Administration, Ministry of Health, Kuwait City 13001, Kuwait; dnmalq@gmail.com; 8 Joint Research Unit in Nutrition and Food, RDC-Nutrition AFRA/IAEA, Biology and Health Laboratory, Ibn Tofail, University-CNESTEN, Kenitra 14000, Morocco; aguenaou.hassan@uit.ac.ma; 9 Programme National de Nutrition, Ministère de la Santé, Rabat 10090, Morocco; elammarilaila0@gmail.com; 10 Regional Office for the Eastern Mediterranean (EMRO), World Health Organization (WHO), Cairo 11371, Egypt; janajabbour@gmail.com (J.J.); aljawaldeha@who.int (A.A.-J.)

**Keywords:** adults, nutrients adequacy, nutrition transition, trajectory: food balance sheet, EMR

## Abstract

The Eastern Mediterranean Region (EMR) is experiencing a nutrition transition, characterized by the emergence of overnutrition and micro-nutrient deficiencies. No previous study has comparatively examined nutrient intake in adults across countries in the EMR. This review examined the adequacy of nutrients in adults living in the EMR. Moreover, it analyzed the food balance sheets (FBS) for 1961–2018 to identify the trajectory of energy supply from macro-nutrients in the EMR. A systematic search was conducted from January 2012 to September 2020. Only observational studies were retained with a random sampling design. An assessment of the methodological quality was conducted. Levels of nutrient daily intake and their adequacy compared to the daily reference intake of the Institute of Medicine were reported across the region. No studies were identified for half of the region’s countries. Although nutrient energy intake was satisfactory overall, fat and carbohydrate intake were high. Intake of vitamin D, calcium, potassium, zinc, and magnesium were below that recommended. The analysis of the FBS data allowed for the identification of four linear patterns of trajectories, with countries in the EMR best fitting the ‘*high-energy-supply from carbohydrate*’ group. This systematic review warrants multi-sectorial commitment to optimize nutrient intake.

## 1. Introduction

The burden of non-communicable diseases (NCDs) has increased significantly in low- and middle-income countries in the last decades, with unhealthy diet being a major risk factor [1,2]. The Global Burden of Disease 2017 collaborators identified the leading nutritional risk factors of death and disability (high intake of sodium, low intake of whole grains and low intake of fruits) to be responsible for the deaths of 3 million (95% Confidence Interval (CI): 1–5), 3 million (95% CI: 2–4) and 2 million (95% CI: 1–4) individuals worldwide, respectively [3,4].

Concomitantly with the emergence of chronic NCDs, several countries from the Eastern Mediterranean Region (EMR) have undergone a nutrition transition in the last decades [5]. This nutrition transition was characterized by dramatic changes in nutrient intakes, specifically an increase in energy, salt, sugar and fat intakes (total fat, saturated fatty acids (SFA) and trans-fatty acids (TFA)) [4]. Although the nutrition transition was found to be associated with increased consumption of energy-dense, micro-nutrient-depleted foods [6], no systematic review has been conducted to document this nutritional phenomenon in the EMR. However, proxy measures of the nutrition transition have been assessed and revealed the co-occurrence of micro-nutrient deficiencies and the alarming increases in the rates of overweight and obesity among all age groups [7]. There is an urgent need to thoroughly assess nutrient intake in the EMR to inform policy and decision makers, given the lack of updated information.

The food balance sheet (FBS) by the Food and Agriculture Organization is used to track the macro-nutrient availability over time and measure the food and macro-nutrient availability at the national level, yet it does not provide a consumption assessment by households or individuals. The FBS can be analyzed through group-based trajectory modeling to understand the changes in consumption patterns over time and to identify subgroups within a population that follow distinctive trajectories [8,9]. To our knowledge, this approach has not been applied to analyze FBS. In view of the current research gaps, this meta-analysis systematically reviewed the literature to address three objectives: (1) to synthesize the available evidence-based reporting on dietary intakes (macro-nutrient, minerals, trace elements and vitamins) of adults in the EMR, (2) to assess the intake adequacy of these nutrients in the region, and (3) to identify the trajectory of energy supply from macro-nutrients for the period of 1961 to 2018 in the EMR.

## 2. Methods

### 2.1. Search Strategy

To identify dietary intake data based on individual food consumption surveys, a systematic database search was carried out to identify relevant studies conducted from 1 January 2012 to 30 September 2020. Studies wrote in English, French and Arabic languages were included in the review.

The PubMed (via NCBI) and Web of Science databases were selected. The search terms for PubMed were: (“dietary pattern” OR “diet quality” OR “food habits” OR “nutrition surveys” OR “diet surveys” OR “food-frequency questionnaire” OR “diet records”) AND (“Afghanistan” OR “Bahrain” OR “Djibouti” OR “Egypt” OR “Iran” OR “Iraq” OR “Jordan” OR “Kuwait” OR “Lebanon” OR “Libya” OR “Morocco” OR “Oman” OR “Pakistan” “Palestine” OR “Qatar” OR “Kingdom of Saudi Arabia” OR “Somalia” OR “Sudan” OR “Syria” OR “Tunisia” OR “United Arab Emirates “ OR “Yemen”).

The search terms for Web of Science were: (Topic Search = ((“dietary pattern” OR “diet quality” OR “food habits” OR “nutrition surveys” OR “diet surveys” OR “food-frequency questionnaire” OR “diet record” OR “dietary recall”)) OR Title = ((“dietary pattern” OR “diet quality” OR “food habits” OR “nutrition surveys” OR “diet surveys” OR “food-frequency questionnaire” OR “diet record” OR “dietary recall”))) AND CU = Country (all aforementioned countries were used in a separate sequence).

### 2.2. Inclusion and Exclusion Criteria

The inclusion and exclusion criteria created for the selection process are listed in Table 1. Data from the same cohort studies were included only if the dietary assessment was carried out at a different time or for a different age group.

### 2.3. Adequacy of Energy and Nutrient Intake

The recommended daily intakes (RDIs) set by the Institute of Medicine were used to evaluate the adequacy of nutrient intakes [6,10]. The upper limit of 2000 mg per day was used to appraise the adequacy of sodium intake, as recommended by the World Health Organization (WHO) [7]. For energy, polyunsaturated fatty acids (PUFA), monounsaturated fatty acids (MUFA), and SFA, we used the RDI for the French population [11]. If the per sex-based analysis was not provided in the study, the RDI was calculated using the following formula, where the RDI was sex-specific:

(1) $$\left[\left({\mathrm{DRI}}_{\mathrm{Male}}\times \mathrm{Male}\;\mathrm{percentage}\right)+\left({\mathrm{DRI}}_{\mathrm{Female}}\times \mathrm{Female}\;\mathrm{percentage}\right)\right]÷100$$

The adequacy of the diet was also assessed by comparing the macro-nutrient distribution ranges to the following cut off points: 10–15% of energy from protein, 15–30% from total fat, and 55–75% from carbohydrate [7].

### 2.4. Data Extraction and Meta-Analysis

The review was conducted in line with the Preferred Reporting Items for Systematic review and Meta-Analysis Protocols [12] and the Meta-analyses of Observational Studies in Epidemiology [13] checklists. The article quality check was assessed using the National Institutes of Health study quality assessment tool for observational cohort and cross-sectional studies [14]. The following data were extracted: (i) paper and participant characteristics (country, first author name, number, age, gender; method of dietary assessment), (ii) the average daily energy value, and (iii) the average daily intake of nutrients (protein, carbohydrate, dietary fibers, total fat, SFA, PUFA, MUFA), minerals, trace elements and vitamins. Means (M) and standard deviations (sd) or standard errors of the mean (SEM) for energy and nutrient intake were collected.

### 2.5. Energy and Macro-Nutrient Supply

The FBS data was evaluated for the period of 1961–2018 based on the old and new methodologies [15,16]. In 2013, methodological modifications were introduced for the computation of the FBS. In the old version, losses were calculated based on non-updated loss ratios and stocks were estimated as residuals from the balance of domestic utilization and domestic supply [17]. For the purpose of our study, all available data were assessed separately from the old [15] and new FBS [16]. Two files were merged to cover the period of 1961–2018. We averaged the intake over ten years except for the period of 1961–1969 and 2000–2018. The following formulas were used to generate the different variables (percentage of energy supply from macro-nutrients) derived from the food item supply data for the period of 1961–2018.

$$\%\;\mathrm{Energy}\;\mathrm{supply}\;\mathrm{from}\;\mathrm{Protein}=\frac{\mathrm{Protein}\;\mathrm{supply}\;\left(\mathrm{kcal}\right)}{\mathrm{Dietary}\;\mathrm{energy}\;\mathrm{supply}\;\left(\;\mathrm{kcal}\right)}\;\times \;100$$

$$\%\;\mathrm{Energy}\;\mathrm{supply}\;\mathrm{from}\;\mathrm{Carbohydrates}=\frac{\mathrm{Carbohydrate}\;\mathrm{supply}\;\left(\;\mathrm{kcal}\;\right)}{\mathrm{Dietary}\;\mathrm{energy}\;\mathrm{supply}\;\left(\;\mathrm{kcal}\right)}\;\times \;100$$

$$\%\;\mathrm{Energy}\;\mathrm{supply}\;\mathrm{from}\;\mathrm{Lipids}=\frac{\mathrm{Lipid}\;\mathrm{supply}\;\left(\mathrm{kcal}\right)}{\mathrm{Dietary}\;\mathrm{energy}\;\mathrm{supply}\left(\mathrm{kcal}\right)}\;\times \;100$$

In order to assess nutrient availability changes over several decades with the changing age-structure of the population, the values were adjusted by dividing them by the number of adult male-equivalents. This number is calculated using a Food and Agriculture Organization intake scale, in which an adult man counts for 1 adult-equivalent. For simplicity, we present only men trajectories.

### 2.6. Data Analysis

#### 2.6.1. Variables Handling

For studies with average categorized daily intake (e.g., by region, age or socioeconomic level), the overall sample intake was computed by directly averaging the values. However, when the different categories did not display the same sample size, the contribution of each category for the overall mean was weighted by its size. If only the adequacy of nutrient intake was reported, we estimated the daily nutrient intake based on the daily recommended intake (provided by the Institute of Medicine [6,10]) for the average age of the studied population. Subsequently, the standard error of the mean was imputed by a value reported in another study, which was preferentially conducted in the same country, having the nearest sample size and level of nutrient intake, and using the same dietary method where possible [18]. All SD values were converted to SEM by using the following formula:

$$SEM=\frac{SD}{\sqrt{N}}$$

where *N* is the sample size.

Finally, when a study included two or more dietary assessment methods, the 24-h dietary recall was privileged. Otherwise, data from a food frequency questionnaire or average of repeated measurements were used. Daily nutrient intake was expressed as a weighted mean with 95% confidence interval (95% CI). Adequacy of intake was expressed as variance weighted percentage.

For statistical analysis, the average age was used or the median value if only the age interval of the assessed population was available.

#### 2.6.2. Meta-Analysis

The continuous variables were analyzed with the ‘*Metan*’ command of STATA 16.1 (StataCorp. 2019. StataCorp LLC: College Station, TX, USA) [19]. The means were used as effect size statistics [20]. The inverse-variance–weighted meta-analysis was used to weigh these effect sizes. The meta-analysis was conducted collectively for all countries, using a random-effects meta-analysis as it accounts for study heterogeneity (e.g., culture, methods, sample size). The heterogeneity of the studies was assessed using the I^2^ statistic applied to the estimated size means, with values greater than 70% indicating substantial heterogeneity [18]. We used a simple linear regression model adjusted for age, sex-ratio and the dietary assessment method for the comparison of countries’ level of nutrient intake, when data was available for at least five countries.

Trajectories of macro-nutrients: we used group-based multi-trajectory modeling, which allows for the assessment of nutrient co-development trajectories, the evaluation of the adequacy of macro-nutrient supply considering their possible inter-relationships and the classification of countries based on their levels of macro-nutrient supply [9]. To be able to run this analysis, data of all countries of the FBS database were used. We fit up to six models and evaluated their adequacy based on the Bayesian information criterion (BIC) and sample size adjusted BIC and Akaike’s information criterion. With regard to sample size, six latent classes were tested in order to preserve parsimony. Since data was available for several decades, we examined the polynomial order of each latent class to determine the pattern of change over years (linear, quadratic, or cubic). Validation of the model was based on a minimum mean posterior probability of the country belonging to its respective group ≥70%, odds of correct classification ≥5 and that each group included ≥5% of countries in the sample. The final validation was carried out based on a visual appreciation of plots and the significance. Each group was labeled in order to mirror their patterns of change for their contribution of macro-nutrients to the daily energy supply.

## 3. Results

**Figure 1:**
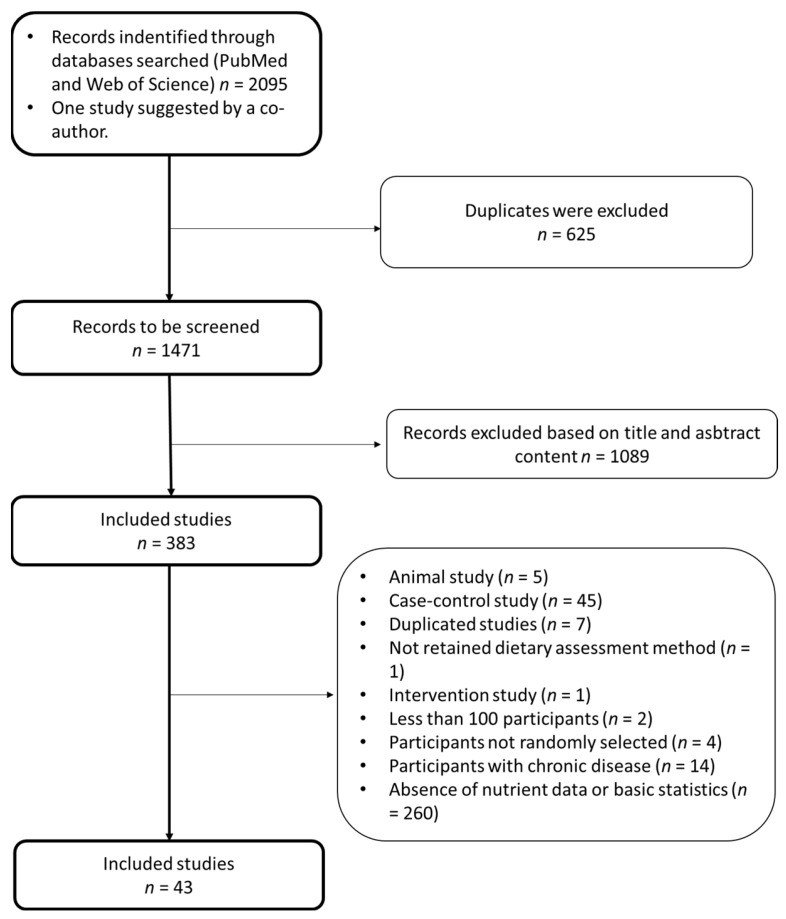
QUORUM: Micro- and macro-nutrient intake among adults in the Eastern Mediterranean Region: a meta-analysis.

The data availability by country is presented in Supplementary Table S1. After screening 2095, 43 studies (*n* = 72,534 subjects) were included (Figure 1).

The overall study characteristics are displayed in Table 2 [21,22,23,24,25,26,27,28,29,30,31,32,33,34,35,36,37,38,39,40,41,42,43,44,45,46,47,48,49,50,51,52,53,54,55,56,57,58,59,60,61,62]. Iran was the most represented country (*n* = 55,592, 76.6%). The mean age was 37.5 ± 8.7 years and women were predominant, with a gender-ratio of men:women of 0.7. Some studies included older subjects, such as Abshirini et al. [21] and Benhammou et al. [51], where the mean age was 56 years and 42 years, respectively.

The total number of participants ranged between 100 and 9809. Only one study used the 3-day food record method [56], and most of the dietary assessments were realized using the food frequency questionnaire (67.4%). Most of the studies (81.3%) were conducted in middle-upper income level countries, namely Jordan, Iran, Lebanon, and Libya. The studies’ quality check is reported as Supplementary Material (Table S1).

### 3.1. Energy Intake

Among 22 countries of the EMR, data on energy intake was found on 11 countries only. The average daily intake was 2287 kcal (95% CI: 2186–2388) and ranged from 1805 kcal (95% CI: 1628–1982) in the Kingdom of Saudi Arabia (KSA) to 2870 kcal (95% CI: 2757–2895) in Libya (Table 3). The adjusted regression analysis showed a significant difference across countries (*p* = 0.011). By contrasting levels of intake of countries, we found that participants from Libya had a higher daily intake than those from Pakistan (*p* = 0.036). Participants from Iran had a higher intake than those from the KSA (*p* = 0.024). Adults in Lebanon had a higher intake compared to those from the KSA (*p* = 0.029). Excess of energy intake was found in Libya (143.5% of RDI) while in the KSA, the intake covered only 74.1% of individuals’ needs.

### 3.2. Macro-Nutrient Intakes

#### 3.2.1. Protein Intake

The analysis showed that protein intake amongst adults in the EMR was more than sufficient (161.5%) and contributed up to 13.9% of the total energy intake, despite some inter-country variations (Table 3). For example, in KSA, 18.9% of daily energy intake was derived from proteins among adults compared to 11.8% among Lebanese adults. Consistently, the regression analysis supported the inter-country difference (*p* < 0.0001). Out of 11 countries, six showed a contribution of protein intake exceeding 15% of energy intake.

#### 3.2.2. Total Carbohydrate Intake

Carbohydrate average intake was 2.3 times higher than the RDI (Table 3). All countries displayed a high intake of carbohydrate, ranging from 175.7% of RDI in Kuwait to 291.8% in Tunisia. In Kuwait, Lebanon, Libya, United Arab of Emirates, the carbohydrate contribution to daily energy intake fell below 50%. The regression analysis revealed a significant difference across countries (*p* = 0.016) for the average daily intake of carbohydrate.

#### 3.2.3. Dietary Fibers

On average, daily intake of fibers represented 72.9% of the RDI (Table 4). A significant difference was found between countries (*p* = 0.042).

#### 3.2.4. Total Fat Intake

Across the region, the total fat intake slightly exceeded the limit of 30% of daily contribution to the energy intake. Adults from the KSA displayed the lowest coverage of 59.6%. The highest intake was in Libya with an intake representing 144% of RDI (Table 3). Seven countries out of eleven showed a high contribution of fat to energy intake (up to 35.5% in Morocco). The regression analysis showed a significant inter-country difference for total fat intake (*p* < 0.0001).

#### 3.2.5. Fatty Acids Intake

Average SFA intake was estimated at 29.0 g/d (95% CI: 24.9–33.0), which is 57% higher than the requirements (Table 4). The highest intake was in Morocco (241.8% of RDI) and the lowest in Tunisia (92.3% of RDI). Intake of MUFA was found to be very low across all countries with an average intake of 67.5% of RDI. A reasonable level of intake was shown in Morocco (106.9%) and Lebanon (81%), while a very low intake was found in the United Arab Emirates, with individuals meeting on average 54.5% of their requirements. The PUFA intake was very high across all countries (>120% of RDI).

#### 3.2.6. Macro-Nutrient Intake by Gender

Data on intake by gender were available from eight countries and nine studies. Overall, men had a more adequate intake in regard to energy intake (78.9% of RDI vs. 69.1% of RDI for women). Men consumed 2 times the RDI for carbohydrate and protein, while women achieved approximately 1.5 times the RDI for these macro-nutrients. A similar level of adequacy for total fat intake was found for both genders (79.5% for men vs. 76.5% for women).

### 3.3. Adequacy of Minerals Intake

**Figure 2:**
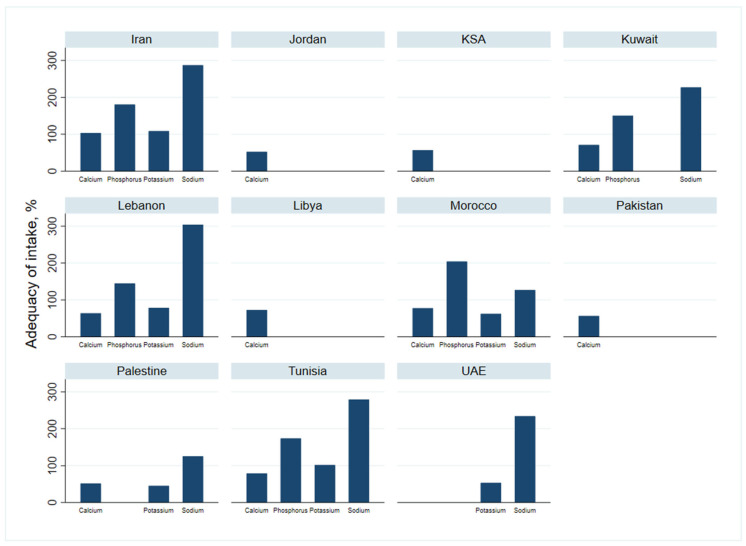
Adequacy of minerals intake for people in the countries sampled in the Eastern Mediterranean Region.

The daily mean intakes of minerals are reported in Table 4 and adequacy of intakes in Figure 2.

#### 3.3.1. Calcium Intake

There was a significant difference in calcium intake between countries (*p* = 0.020). Calcium intake was found to be inadequate (74.1% of the RDI) in all countries except in Iran (103.2%). The lowest level of intake was found in Palestine (51.7%).

#### 3.3.2. Potassium Intake

Data for potassium was available for five countries. The average intake was 82.4% of the RDI. The highest potassium daily intake was found in Iran: 3226 mg/d (95% CI: 2257.0–4196.9), equivalent to 108.7% of the RDI, while the lowest intake was observed in Palestine (1242.1 mg/d (95% CI: 1163.1–1321.1), which is equivalent to 45.3% of the RDI). Palestine was followed by Morocco with an average intake of 1863 mg/d (95% CI: 1784–1942), corresponding to only 62.2% of the daily requirements.

#### 3.3.3. Sodium Intake

Sodium intake varied across countries (*p* = 0.006). The average daily intake of sodium in all countries was 3651.6 mg/d (95% CI: 3325.5–3977.6), 2.5 times greater than the recommended levels set by the WHO. Daily intake ranged between 1901 mg (95% CI: 1714–2087) in Morocco to 4562 mg (95% CI: 4488–4635) in Lebanon.

#### 3.3.4. Phosphorus Intake

Data was found for five countries only. The average intake was above the requirements in all countries. The average intake for all countries was about 170%, which ranged between 144.9% in Lebanon to 204% in Morocco.

### 3.4. Adequacy of Trace Elements Intake

**Figure 3:**
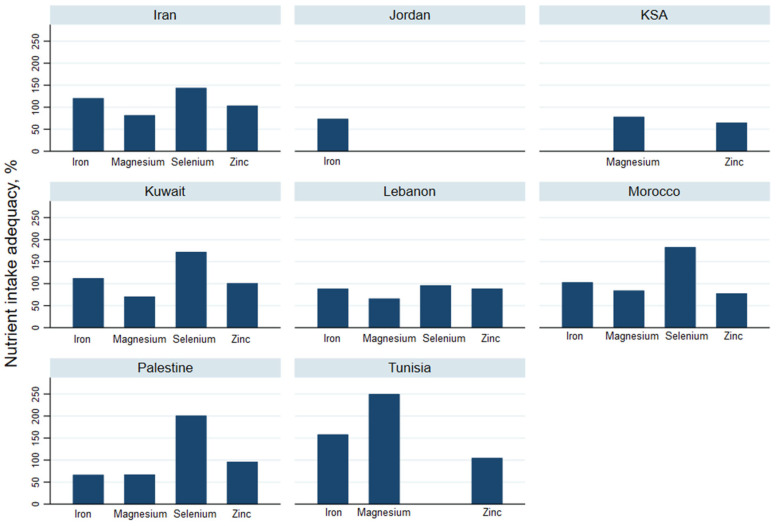
Adequacy of trace elements intake for people in the countries sampled in the Eastern Mediterranean Region.

Table 4 and Figure 3 summarize trace elements intake and adequacy.

#### 3.4.1. Iron Intake

A significant difference was found in iron intake across countries (*p* = 0.0005). Nearly all countries (except Jordan) reported a fairly acceptable level of daily iron intake ranging from 10.8 mg (95% CI: 9.5–12.1) in Palestine to 19.5 mg (95% CI: 18.8–20.2) in Tunisia, corresponding to 66.4% and 158.3% of RDI, respectively. The iron intake in Lebanon and Jordan was low, amounting to between 73.5% and 88.5% of needs, respectively.

#### 3.4.2. Selenium and Zinc Intakes

Data on selenium and zinc intake was available in four countries only. Their intake was sufficient overall and ranged between 96.4% in Lebanon to 200.9% in Palestine. Similarly, zinc intake was satisfactory with 92.1% of the RDI across studied countries. However, Saudi adults showed a very low intake of 64.8% followed by Morocco with a mean intake of 78.0% of the RDI.

#### 3.4.3. Magnesium Intake

A significant difference (*p* = 0.010) was depicted for magnesium intake across studied countries. The highest intake was found among Tunisian adults (245.3% of the RDI) and the lowest level of intake was found in Lebanon (66.0% of the RDI).

### 3.5. Adequacy of Vitamins Intake

#### 3.5.1. Vitamin A, D and E

**Figure 4:**
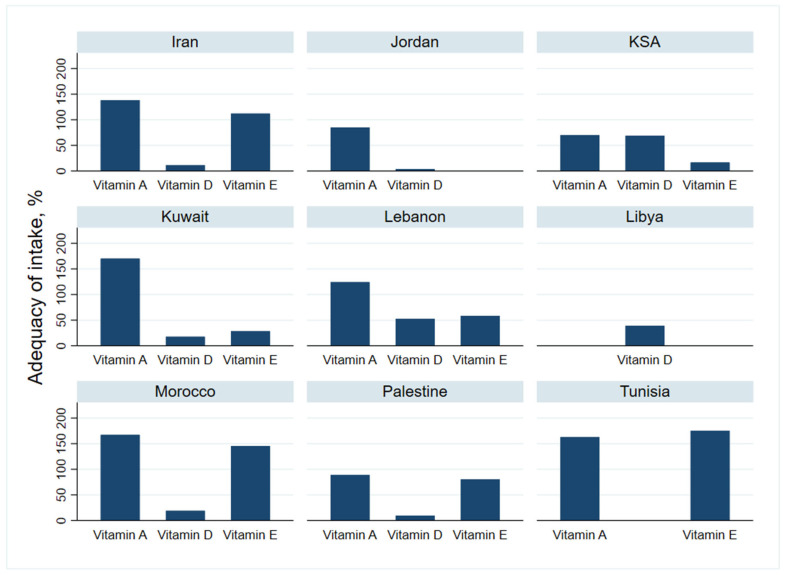
Adequacy of liposoluble vitamins intake for people in the countries sampled in the Eastern Mediterranean Region.

Data on vitamins A, D and E intake are presented in Figure 4 and Table 4.

##### Vitamin A Intake

Data on Vitamin A, collected from six countries, showed no significant difference in intake (*p* = 0.051). Vitamin A intake ranged between 69.8% in KSA to 170.1% in Kuwait. The mean daily intake was 708.4 µg/d (95% CI: 480.6–936.2), corresponding to 129.4% of the RDI.

##### Vitamin D Intake

The average vitamin D intake was very low with an average daily intake of 3.1 µg (95% CI: 2.1–4.1), covering less than 70% of RDI.

##### Vitamin E Intake

The level of intake was equal to 90.7 and ranged from 28.3% of the RDI in Kuwait up to 175.3% of the RDI, in Tunisia.

#### 3.5.2. Water Soluble Vitamins

**Figure 5:**
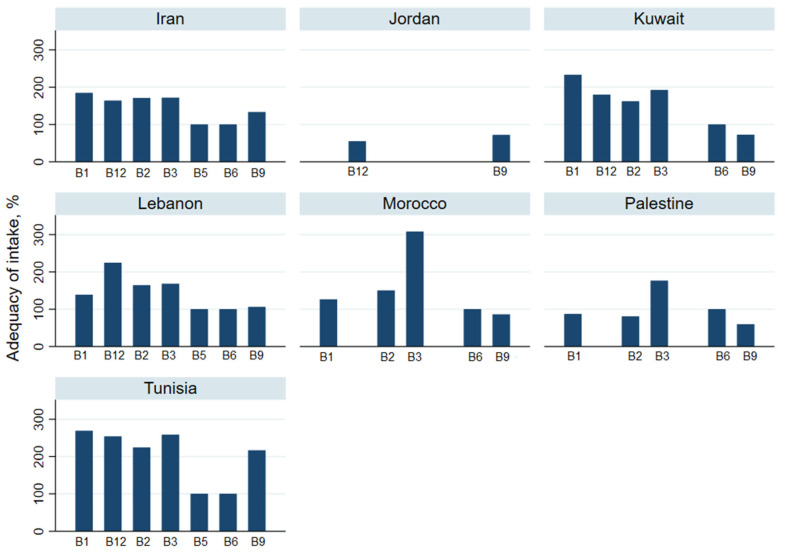
Adequacy of vitamins B intake for people in the countries sampled in the Eastern Mediterranean Region.

The adequacy of B vitamin intake is displayed in Figure 5 and Table 4. Daily Vitamin B1 intake ranged between 1.2 mg (95% CI: 1.1–1.3) in Morocco to 2.5 mg (95% CI: 2.5–2.6) in Kuwait; vitamin B2 from 0.7 mg (95% CI: 0.6–0.8) in Palestine to 2.2 mg (95% CI: 2.1–2.3) in Tunisia; Vitamin B3 from 19.1 mg (95% CI: 18.4–19.8) in Lebanon to 35.4 mg (95% CI: 34.6–36.1) in Morocco; Vitamin B9 from 191.4 μg (95% CI: 188.2–194.6) in Palestine to 692.7 μg (95% CI: 680.4– 705.1) in Tunisia; and vitamin B12 from 1.10 μg (95% CI: 1.07–1.12) in Jordan to 5.1 μg (95% CI: 4.8–5.3) in Tunisia. Overall, intake was satisfactory except among adults from Jordan (for vitamin B9 and B12) and Kuwait (for vitamin B9).

Data for vitamin B5 was reported only among three countries, and the daily average intake was 4.8 µg (3.3–6.3). Overall, the intakes met 82.4% of the RDI. The vitamin B6 intake was 1.5 times or higher than the RDI among all countries (Iran, Kuwait, Lebanon, and Morocco).

For vitamin C, data was not available for Libya, Pakistan, and United Arab Emirates. Overall, the intakes were 1.7 times higher than the RDI, ranging from 49% in Jordan to 524% in Tunisia. Accordingly, the regression analysis showed a significant difference across countries (*p* < 0.0001).

### 3.6. Contribution of Macro-Nutrients to the Trajectories of Energy Supply

**Figure 6:**
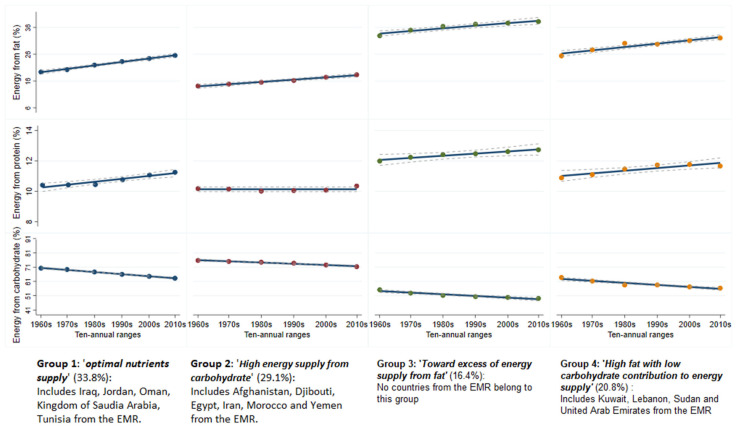
Countries’ profile of dietary energy supply from macro-nutrients (1961 to 2018). Figure caption: Dashed lines refer to 95% confidence interval. *X*-axis label: 1960s refers to 1961 to 1969; 1970s refers to 1971–1979; 1980s refers to 1980–1989; 1990s refers to 1990–1999; 2000s refers to 2001–2009; 2010s refers to 2010–2018.

The analysis of the FBS data allowed for the identification of four linear patterns of trajectories (Figure 6). The most important group (33.8%) was labelled ‘*optimal nutrients supply*’, described as an increasing energy supply from fat and protein and a decreased energy supply from carbohydrates. Irrespective of the trends, the contribution of macro-nutrients to the dietary energy supply was within the WHO recommended ranges. The first group included Iraq, Jordan, Oman, KSA and Tunisia from the EMR. The second group (29.1%), labeled ‘*High energy supply from carbohydrate*’, captured countries where the contribution of carbohydrate to the dietary energy supply was high, yet declining gradually. This was the only group with a stable level of energy supply from protein that barely met individuals’ needs. This group included six countries from the EMR, namely Afghanistan, Djibouti, Egypt, Iran, Morocco and Yemen. The third group (20.8%) was labelled ‘*Toward excess of energy supply from fat*’ and describes countries with an acceptable level of energy supply from fat until the mid-1980s (1980–1989), followed by a significant and continuous increase until 2018. No country from the EMR belonged to this group. The smallest group (16.4%) was labelled ‘*High fat with low carbohydrate contribution to energy supply*’ and described trajectories of countries with a high fat contribution to the dietary energy supply concomitantly with a low contribution of carbohydrate, crossing the lower bound of 50% recommended by the WHO. This group included Kuwait, Lebanon, Sudan (former) and the United Arab Emirates.

## 4. Discussion

This is the first meta-analysis to report on the intakes of micro- and macro-nutrients among adults in the EMR. Data was found for 50% of the countries from the region. The situation identified a typical region facing a nutritional transition. Results indicated that energy, protein, carbohydrate, and fat intakes exceeded the recommendations. Consistently, inadequate intakes of calcium and potassium were observed, along with an elevated sodium intake. Trace elements’ intake seemed to be less problematic, as on average 80% or more of the requirements were met. Remarkably, inadequate vitamin D intake was observed in all studies while for other vitamins, such as B vitamins, intakes ranged from adequate to excessive, without reaching the upper intake limits.

The available data provided evidence that nutritional intake among adults in the EMR has been challenged with high consumption of macro-nutrients (carbohydrates, total fats, and proteins in terms of crude quantity and/or as contributors to the total energy intake), and of empty calories coming from carbohydrate-rich, fiber-poor sources. For example, the carbohydrate consumption was over 200% RDI for most nations and countries like Jordan, Kuwait, Lebanon and Tunisia displayed a low dietary fiber consumption. While SFA intake was found to be high in countries such as Iran, Jordan, Lebanon and Morocco, it was low in Tunisia. Sodium intake was very high, exceeding the WHO recommended levels in all assessed countries of the EMR. The deficient intake of potassium seen in most countries is expected to enhance the harms of excess sodium intake [63]. Exceptionally, adults from Iran showed a sufficient level of calcium intake. Marked and common intake deficiencies were found for magnesium, vitamin D and vitamin E. Given the deficient vitamin D intake and widespread serum vitamin D deficiency documented recently in the EMR [64], there is currently enough evidence to recommend systematic vitamin D supplementation among adults in the EMR to meet RDIs.

Because the included studies cross-sectionally assessed nutrient adequacy, we tried to compensate for this limitation by appraising trajectories of macro-nutrient contribution to the dietary energy supply based on the FBS data. The countries of the EMR belonged to three of the four identified profiles. Iraq, Jordan, Oman, KSA and Tunisia belonged to the ‘*optimal nutrients supply*’ group, which might mirror somewhat the effort done during the last decade at the macro-level towards improving the population diet. Nine countries were classified as ‘*high energy supply from carbohydrate*’ or ‘*toward excess of energy supply from fat*’, supporting the meta-analysis findings. According to the conducted analysis, the nutrition transition is not at the same stage in all countries. This observation is consolidated by longitudinal analysis of macro-nutrient supply trajectories derived from the Food and Agriculture Organization data, as most countries have inadequate levels of supply. Countries’ heterogeneity is expected as the EMR hosts countries with different income levels, food accessibility and food security levels. The paradoxical nutritional situation is evident with countries experiencing an alarmingly increasing rate of obesity while others struggle with undernutrition [65]. Diet could be influenced by several factors at the macro-, meso- and micro-level. The difference reported between countries could be a consequence of macro-level factors (macro-environment) referring to structures such as food systems (access to land and food production), nutrition policies and reforms, mass media and culture. The macro-environment exerts an influence on micro-level factors, which in turn would influence the population’s diet.

In the EMR, the suboptimal nutrient profile of the adult population has been associated with an increase in diet-related NCDs. This is problematic, especially among women of reproductive age, as it might drive the inter-generational cycle of malnutrition. The nutrition transition in the region is not restricted only to the shift occurring in nutrients intake but also extends to the increasing level of physical inactivity. Indeed, EMR has the second highest prevalence of insufficient physical activity (34.9%) in the world [66]. In some countries like Kuwait and KSA, the level of insufficient physical activity reaches 67% and 53.1% [66], respectively. For sustainable change to happen, reliable data and regular assessments are essential to be able to inform and drive nutrition action and assess progress towards the agreed targets. It is also important for countries of the EMR to have the capacity to analyze and interpret the information, in order to be able to communicate effectively with decision-makers. Robust integrated multisectoral and sustainable information systems in the dietary sectors are needed to inform and improve policy development and provide accountability. Such systems need technical and financial support and could benefit from countries working together in sub-regional groupings to learn lessons from one another’s experience. The WHO Regional Office for EMR developed a strategy in nutrition that supports countries towards achieving the existing global nutrition and NCD goals, which have been incorporated into the sustainable development goals. In order to monitor progress, a Global Nutrition Monitoring Framework has been established, which defines the indicators for the global nutrition targets. Similarly, countries are already tracking towards the NCD goals through the Global Monitoring Framework on Noncommunicable Diseases. Such reliable information systems are needed in all countries to enable them to report on the progress towards global targets for both nutrition and NCDs.

Dietary guidelines provide evidence-based recommendations on food choices to meet nutrient requirements. Indeed, a comparative dietary regimen from around the world showed that the closer to whole food plant-based nutrition we are, the better the health outcomes, quality of life, and reduction in disability and premature death (See EAT Lancet Commission, 2019 [67]). The so-called ‘planetary heath diet’ emphasizes the consumption of whole grains, fruits, vegetables, nuts and legumes, while meat and dairy are still considered important parts of the diet but in smaller proportions than the plant portion. The commission recommended that sugar should be cut in half. Specific daily targets were established for each food group, e.g., 232 g for whole grains, 300 g for vegetables, 200 g for fruits. In line with the Eat-Lancet commission, we examined closely how ‘protein’, in particular, was assessed in the retained studies, and whether due weighting was given to ‘plant-based’ protein sources. Unfortunately, all studies reported protein intake as a total or as a contribution to the daily energy intake and none of them focused on protein source (vegetable vs. animal). Hence, more focus should be put on the importance of plant-derived proteins for health promotion.

A strength of this study is that it is a systematic literature review of studies conducted during the last decade across all countries of the EMR. The included studies were subject to a quality check based on internationally approved tools. The weighting of statistical analyses by variance and the inclusion of more than one study for some countries increased the external validity of our meta-analysis. Moreover, the use of a group-based multi-trajectory technique allowed for the consideration of the interrelationship of multiple nutrients and for the identification of trajectory patterns of energy and macro-nutrients over six decades. This work is subject to some limitations. Data for only eleven countries were found, which may limit statistical inference to the whole region. Due to the unavailability of country-specific recommendations of nutrient intakes, we used the Institute of Medicine RDI. This might have introduced a non-negligible bias to our interpretation of level of nutrient intake adequacy. Furthermore, it is worth mentioning that US standards might not be a suitable target for people living in the EMR in order to reduce the burden of NCDs. Indeed, the US is rife with NCDs and population health risks related to nutrition, particularly extensive consumption of ‘processed, ultra-processed, and hyper-palatable foods’. The included studies used a mixture of dietary assessment tools, which might have introduced a measurement bias. The used food frequency questionnaire included different food items and the 24 h dietary recall was not always conducted over three non-consecutive days. Finally, we expect the interpretation of nutrient intake adequacy to be biased by the missing data in the food composition tables in the region.

## 5. Conclusions

This first meta-analysis assessing nutrients adequacy in the EMR revealed that countries from the EMR did not meet RDI for most nutrients. Lack of national representative data was clearly reflected among adults and missing data could be more pronounced among other specific groups such as pregnant women. The meta-analysis results reflect the nutrition transition occurring in the region, especially with the excess intake of fat, carbohydrate and sodium, along with the deficient intake of certain trace elements (e.g., zinc and magnesium) and vitamins (e.g., vitamin D). Health policies should focus on setting up sustainable food systems that facilitate healthy and sustainable food choices [68] and to continue the promotion of healthy dietary patterns to optimize the nutrient intakes in order to meet the requirements. Moreover, in view of the data scarcity in the EMR, establishing and/or strengthening nutrition surveillance systems to assess the nutrition status of populations is strongly recommended for all countries of the region. An update and expansion of the culturally specific food composition databases, with standardized methodology and reporting in line with WHO recommendations, is key for conducting dietary intake studies and for the generation of reliable assessments of micro- and macro-nutrients intake.

## Figures and Tables

**Table 1:** Inclusion and exclusion criteria for the selection of studies.

	Inclusion Criteria	Exclusion Criteria
Setting	Any country from the EMR as defined by the WHO	Countries from other regions as defined by the WHO
Year of publication	From January 2012 to September 2020	Before 2012 and after November 2020
Age	Average or median age between 19–64 years	Average or median age < 19 y or ≥ 65 years.
Sample size	Studies with ≥100 participants	Studies with <100 participants
Language	Studies published in English, French or Arabic.	Studies published in languages other than English, French or Arabic
Participant characteristics	Only health subjects were retained from random sampling studies	Unhealthy participants from non-random sampling studies
	Any socioeconomic level	Pregnant women
Study design	Cross-sectional or longitudinal studies	Studies including only participants suffering from a disease
		Studies in duplicate (different papers using data collected during the same wave)
		Interventional studies
		Clinical trial studies
		Case-control studies
		Studies that did not include statistics analysis (did not present means, standard deviations/standard errors)
Representativeness	National, regional or district	

EMR: Eastern Mediterranean Region, WHO: World Health Organization.

**Table 2:** Socio-demographic characteristics of studies included in the systematic review [21,22,23,24,25,26,27,28,29,30,31,32,33,34,35,36,37,38,39,40,41,42,43,44,45,46,47,48,49,50,51,52,53,54,55,56,57,58,59,60,61,62].

Country	Study	Sample Size	Age (y)	Female, %	Dietary Assessment Tool	Quality Rating
Iran	Abshirini et al., 2019 [21]	393	56	100	FFQ	Fair
Iran	Alipour et al., 2015 [22]	184	22.2 ± 2.2	100	3-day 24 HDR	Fair
Iran	Aminianfar et al., 2020 [23]	6724	36.8 ± 8.08	60.4	FFQ	Good
Iran	Azadbakht et al., 2013 [24]	411	18–28	100	FFQ	Good
Iran	Bahadoran et al., 2012 [25]	1944	19–50	56.8	FFQ	Good
Iran	Banikazemi et al., 2015 [26]	7172	48.6 ± 2.0	62.0	24 HDR	Good
Iran	Esfandiar et al., 2019 [27]	4654	40.6 ± 14.3	25.0	FFQ	Good
Iran	Hashemi et al., 2018 [28]	947	36.6 ± 4.9	100	FFQ	Good
Iran	Heidari et al., 2019 [29]	1922	55.9 ± 10.6	49.6	FFQ	Good
Iran	Hosseinpour-Niazi et al., 2015 [30]	4667	41.65 ± 13.8	55.5	FFQ	Good
Iran	Keshteli et al., 2017 [31]	3979	36.4 ± 7.9	55.1	FFQ	Fair
Iran	Mohammadifard et al., 2017 [32]	796	38.9 ± 11.4	56.7	FFQ	Fair
Iran	Mohammadifard et al. [33]	1618	37.3	49.0	FFQ	Good
Iran	Parvaneh et al., 2014 [34]	226	20–55	51.7	3-day 24 HDR	Good
Iran	Rashidi et al., 2018 [35]	9809	48.33 ± 8.26	40.1	1-day 24 HDR	Good
Iran	Sadeghi et al., 2018 [36]	6583	36.8 ± 8.1	60.3	FFQ	Good
Iran	Safabakhsh et al., 2020 [37]	393	57.2 ± 6.3	100	FFQ	Good
Iran	Zaribaf et al., 2014 [38]	420	35.2 ± 7.2	100	FFQ	Good
Jordan	Tayyem et al., 2014 [39]	101	33.4 ± 18.5	60	3-day 24 HDR	Fair
Jordan	Tayyem et al., 2018 [40]	167	18–51	50	FFQ	Good
Kingdom of Saudi Arabia	Al-Daghri et al., 2015 [62]	185	19–60	53	FFQ	Good
Kingdom of Saudi Arabia	Zareef et al., 2018 [42]	257	20–50	100	FFQ	Good
Kuwait	Zaghloul et al., 2013 [43]	1049	19–50	55.9	One-day 24 HDR	Good
Lebanon	Aoun et al., 2019 [44]	100	18–60	48.0	FFQ	Good
Lebanon	Fahed et al. [60]	286	41.2 ± 11.0	53.1	One-day 24 HDR	Good
Lebanon	Ghadieh R et al., 2018 [45]	344	41.6 ± 11.5	50.0	FFQ	Good
Lebanon	Jomaa et al. [61]	1204	39.6 ± 0.3	100	One-day 24 HDR	Good
Lebanon	Nasreddine et al., 2019 [46]	308	39.79 ± 14.00	62.7	FFQ	Good
Lebanon	Nasreddine et al., 2019 [47]	708	40–59	54.9	FFQ	Good
Lebanon	Nasreddine et al., 2019 [47]	1317	20–39.9	54.9	FFQ	Good
Lebanon	Harmouche-Karaki M et al., 2020 [48]	500	17–64	62.2	3-days 24 HDR	Good
Lebanon	Mansour et al., 2019 [49]	363	39.2 ± 15.2	50.0	FFQ	Good
Libya	Faid et al., 2018 [50]	366	25–64	100	2-day 24HDR	Fair
Morocco	Benhammou et al., 2016 [51]	200	25–70	50.5	FFQ	Good
Morocco	El kinany et al., 2018 [52]	105	27.3 ± 5.6	70.1	24 HDR (repeated 3 times over 4 months)	Good
Pakistan	Iqbal et al., 2014 [53]	144	32.8 ± 11.4	100	24 HDR (repeated 4 times)	Good
Pakistan	Khan et al., 2013 [54]	305	32.0 ± 8.0	100	FFQ	Fair
Pakistan	Parveen et al., 2016 [55]	324	28.9 ± 12.2	100	FFQ	Good
Palestine	Benhammou et al., 2016 [51]	200	25–70	82	FFQ	Good
Tunisia	Abassi et al., 2019 [56]	2545	20–49	35.1	3-day food record	Good
Tunisia	García-Meseguer et al., 2017 [57]	132	21.2 ± 2.8	65.1	2-day 24 HDR	Good
Tunisia	Perignon et al., 2020 [41]	6512	47.4 ± 0.14	51.9	FFQ	Good
United Emirates Arab	Cheikh Ismail et al., 2019 [58]	122	18–25	54.1	One-day 24 HDR	Good
United Emirates Arab	Saber-Ayad et al., 2020 [59]	196	30.3 ± 9.8	55.6	FFQ	Good

24 HDR: 24 h dietary recall; FFQ: Food frequency questionnaire.

**Table 3:** Average daily intake (95% CI) and adequacy of energy and macro-nutrient of people in the countries sampled in the Eastern Mediterranean Region.

Country	Energy		Protein			Carbohydrates			Fat		
	Mean ^1^, 95% CI	Adequacy of Intake ^2^, %	Mean ^1^, 95% CI	Adequacy of Intake, %	% of Energy	Mean ^1^, 95% CI	Adequacy of Intake, %	% of Energy	Mean ^1^, 95% CI	Adequacy of Intake, %	% of Energy
Iran	2166.3 (2046.6–2286.1)	94.3	78.7 (69.8–87.6)	158.4	16.2	299.5 (261.1–337.9)	230.4	55.3	75.5 (67.9–83.1)	91.3	31.4
Jordan	2282.2 (974.5–3589.9)	93.8	76.0 (50.1–101.9)	150.3	13.3	345.8 (115.9–575.7)	266.1	60.6	70.5 (28.2–112.7)	80.4	27.8
Kingdom of Saudi Arabia	1805.0 (1628.4–1981.9)	74.1	85.4 (71.0–99.7)	168.4	18.9	295.9 (293.6–298.2)	227.6	65.5	52.3 (48.2–56.4)	59.6	26.1
Kuwait	2111.0 (2007.1–2214.9)	87.2	67.0 (62.9–71.9)	133.7	12.6	228.4 (211.9–244.8)	175.7	43.3	60.7 (56.5–64.8)	69.6	25.9
Lebanon	2680.9 (2328.7–3033.1)	113.5	79.1 (70.7–87.4)	160.4	11.8	282.6 (258.9–306.2)	216.8	42.2	94.8 (82.9–106.9)	110.1	31.8
Libya	2870.3 (2757.1–2983.5)	143.5	122.2 (118.0–126.4)	265.7	17.0	330.3 (317.4–343.2)	254.1	46.0	105.1 (99.4–110.8)	144.9	32.9
Morocco	1884.6 (1861.5–1907.7)	82.9	83.4 (82.1–84.6)	170.4	17.7	266.2 (158.1–374.4)	201.9	56.5	74.0 (69.6–78.4)	88.9	35.3
Pakistan	1936.3 (1163.2–2709.3)	88.0	56.6 (41.8–71.4)	123.1	11.6	336.1 (331.9–340.3)	258.5	69.4	72.7 (28.8–116.6)	92.1	33.8
Palestine	2228.6 (2205.0–2252.2)	97.3	60.9 (59.9–61.9)	127.5	10.9	341.8 (339.7–344.1)	263.0	61.3	143.4 (138.0–148.8)	174.2	57.9
Tunisia	2419.4 (2086.1–2752.6)	102.1	86.9 (85.4–88.4)	168.3	14.4	379.3 (345.8–412.9)	291.8	62.7	93.7 (67.7–119.7)	110.3	34.9
United Emirates Arab	2663.9 (1060.1–4267.7)	109.3	110.9 (24.0–197.9)	220.9	16.6	308 (118.4–498.7)	234.2	46.2	103.8 (50.2–157.4)	118.7	35.1
Overall	2286.6 (2185.7–2387.9)	98.9	79.9 (74.9–85.1)	161.5	13.9	304.8 (295.4–314.2)	234.5	53.3	82.6 (77.7–87.4)	98.8	32.2

^1^ Weighted mean with 95% confidence interval (CI); ^2^ weighted percentage.

**Table 4:** Average daily intake (95% confidence interval) of micro- and macro-nutrient intakes of people in the countries sampled in Eastern Mediterranean Region.

Parameter	Mean	95% CI	
		Lower CI	Upper CI
Dietary fiber (g/d)	21.8	19.6	24.1
SFA (g/d)	29.0	24.9	33.0
MUFA (g/d)	31.9	25.3	38.6
PUFA (g/d)	22.2	17.4	26.9
Calcium (mg/d)	745.8	656.4	835.2
Potassium (mg/d)	2451.3	1903.2	2999.3
Sodium (mg/d)	3748.0	3384.1	4112.0
Phosphorus (mg/d)	1240.7	1115.6	1365.7
Iron (mg/d)	14.4	12.2	16.6
Selenium (mg/d)	89.7	70.8	108.5
Zinc (mg/d)	8.7	8.0	9.3
Magnesium (mg/d)	382.1	335.9	428.3
Vitamin A (µg/d)	708.4	480.6	936.2
Vitamin D (µg/d)	3.1	2.1	4.1
Vitamin E (µg/d)	10.9	6.7	15.0
Vitamin B1 (µg/d)	1.6	1.2	2.0
Vitamin B2 (µg/d)	1.6	1.2	1.9
Vitamin B3 (µg/d)	24.3	18.9	29.6
Vitamin B5 (µg/d)	4.8	3.4	6.3
Vitamin B6 (µg/d)	1.7	1.4	1.8
Vitamin B9 (µg/d)	380.5	223.3	537.8
Vitamin B12 (µg/d)	3.8	2.8	4.5
Vitamin C (mg/d)	111.4	82.4	140.5

## Data Availability

Not applicable.

## Supplementary Materials

The following are available online at https://www.mdpi.com/article/10.3390/nu13051515/s1, Table S1: Energy and nutrient data availability by country in the Eastern Mediterranean Region.
