# Supplementary material for: Meta-Analysis and Systematic Review of Micro- and Macro-Nutrient Intakes and Trajectories of Macro-Nutrient Supply in the Eastern Mediterranean Region
Source: Nutrients. 2021 Apr 30;13(5):1515. doi: 10.3390/nu13051515 (PMC8145171; doi:10.3390/nu13051515)
Supplement: Supplementary file 1 [file nutrients-13-01515-s001.zip › nutrients-1153690-supplementary.pdf]

Table S1. Energy and nutrient data availability by country in the Eastern Mediterranean Region

	Iran	Jordan	Kingdom of Saudi Arabia	Kuwait	Lebanon	Libya	Morocco	Pakistan	Palestine	Tunisia	United Emirates Arab
Energy	X	X	X	X	X	X	X	X	X	X	X
Protein	X	X	X	X	X	X	X	X	X	X	X
Carbohydrate	X	X	X	X	X	X	X	X	X	X	X
Dietary fibers	X	X		X	X					X	
Total fat	X	X	X	X	X	X	X	X	X	X	X
SFA	X	X			X					X	X
MUFA	X				X		X			X	X
PUFA	X				X		X			X	X
Calcium	X	X	X	X	X	X	X	X	X	X	
Potassium	X			X	X		X		X	X	X
Sodium	X			X	X		X		X	X	X
Phosphorus	X			X	X		X			X	
Iron	X	X		X	X		X		X	X	
Selenium	X			X	X		X		X	X	
Zinc	X		X	X	X		X		X	X	
Magnesium	X		X	X	X		X		X	X	
Vitamin A	X	X	X	X	X		X	X	X	X	
Vitamin D	X	X	X	X	X	X	X		X		
Vitamin E	X		X	X	X		X		X	X	
Vitamin B1	X			X	X		X		X	X	
Vitamin B2	X			X	X		X		X	X	
Vitamin B3	X			X	X		X		X	X	
Vitamin B5	X				X					X	
Vitamin B6	X			X	X		X		X	X	
Vitamin B9	X	X		X	X		X		X	X	
Vitamin B12	X	X		X	X					X	
Vitamin C	X	X	X	X	X		X			X	
